# Sorption of Cadmium and Zinc in Selected Species of Epigeic Mosses

**DOI:** 10.1007/s00128-014-1210-0

**Published:** 2014-01-28

**Authors:** Andrzej Kłos, Ewelina Gordzielik, Małgorzata Anna Jóźwiak, Małgorzata Rajfur

**Affiliations:** 1 Opole University, 6 kard. B. Kominka Str., 45-032 Opole, Poland; 2 Department of Environment Protection and Modelling, Jan Kochanowski University, ul. Świętokrzyska 15, 25-406 Kielce, Poland

**Keywords:** Biomonitoring, Heavy metals, Ion exchange, Sorption properties of mosses

## Abstract

The sorption abilities of seven moss species growing on the area of Bory Stobrawskie forest (southern Poland) were tested in laboratory. Sorption was carried out in solutions of Zn and Cd chlorides. It has been shown that the sorption properties depend on the moss species and increases in the series as follows: *Polytrichum commune* < *Leucobryum glaucum* < *Eurhynchium praelongum* < *Thuidium tamtariscifolium* ≤ *Dicranum scoparium* ≤ *Pleurozium schreberi* < *Sphagnum* sp. With help of microscope images, it was also demonstrated that one of the factors affecting the sorption properties of mosses was the level of their surface development. The determined sorption capacity of Zn varies according to species of mosses from 0.0491 to 0.1287 mmol g^−1^, and in relation to Cd from 0.0319 to 0.1335 mmol g^−1^. The described results may be important in the process of biomonitoring research design and in the test results interpretation.

Mosses, due to their anatomy and the specific type of nutrition, easily absorb substances contained in atmospheric precipitation. On their surface, in crevices and bends, they accumulate dust containing macro- and micro-nutrients, which under favorable conditions dissolve in water that wets the thallus and penetrates its structure. It is estimated that the surface area of *Plagiomnium cuspidatum* and *Taxiphyllum deplanatum* mosses in proportion to dry matter is 1.6 m^2 ^g^−1^ (Darlington et al. 2001). The sorption intensity depends on the type of absorbed substances, including the form in which they occur, size, polarity of molecules or ion charges, and is different for different moss species. The process of cation sorption, based on the ion exchange between the moss thallus and a solution that wets the thallus, is the most known. The ion-exchange process involves several functional groups, including carboxyl groups (–COOH), aldehyde groups (–CHO), hydroxyl groups (–OH) and amino groups (–NH_2_), that form part of cell wall-forming compounds, such as lignin-like phenolic compounds (Sen Gupta et al. 2009). Some authors also point to the possibility of complexing metal ions and the physical adsorption (Ringqvist et al. 2002).

Good sorption properties of mosses, their prevalence, simple identification, year-round availability, population stability and high tolerance to pollutants account for the fact that mosses, along with lichens, have become the most widely used material in biomonitoring of the atmospheric aerosol pollution (Wolterbeek 2002; Markert 2007). Biomonitoring research involves inter alia analyses of chemical composition of mosses and/or lichens collected from their natural habitat (Samecka-Cymerman et al. 2006; Kłos et al. 2010; 2011). Exposure techniques, involving transfer of the biological material from less polluted areas to urban or industrial areas, are also frequently used (e.g. Kłos et al. 2009; Kosior et al. 2010).

Since 1990, many European countries have been conducting cyclic (carried out every 5 years) research on trace elements accumulated in mosses. In 2005, 28 countries, including Poland, participated in the program (Harmens et al. 2010). Similar research was conducted in 2000 in the Visegrad Group countries (Hungary, Czech Republic, Poland and Slovakia) (Suchara et al. 2007). Due to a large extent of biomonitoring research, the above studies incorporate various species of mosses having different sorption properties and thus, in the case of comparative studies, false conclusions can often be drawn.

The aim of the study was to evaluate the sorption properties of seven species of mosses exposed to the presence of xenobiotic (Cd) and trace element (Zn) and to check if in natural conditions different species of mosses absorb the tested metals in accordance with the proportions experimentally determined in the laboratory.

## Materials and Methods

Mosses: *Dicranum scoparium, Eurhynchium praelongum*, *Leucobryum glaucum*, *Pleurozium schreberi*, *Polytrichum commune*, *Sphagnum* sp. i *Thuidium tamtariscifolium*, occurring in Bory Stobrawskie located 20–40 km to the north-west of Opole (Poland) were used in the study.

Green parts of mosses were purified from any mechanical impurities and then washed with demineralised water with a conductivity of κ = 0.5 μS cm^−1^. Mosses, dried at 303 K, were stored in sealed plastic containers.

One g dry mass (d.m.) samples were used for tests. Each species was placed in a perforated 30 cm^3^ container. Containers with mosses were placed for 30 min in demineralized water (2 dm^3^) to remove salts remaining on the moss surface. Then seven containers, with mosses prepared as specified above, were immersed in a 1.4 dm^3^ solution of salt of the analyzed metal (Zn and Cd separately). During the sorption process, the solutions were vigorously stirred. The process had been conducted for 30 min. The studies showed that after 25–50 min. changes in concentrations of analytes in the system could not be determined by the instruments used in measurements. It was assumed that a steady state in the system was achieved. Metals were determined in solutions and in mineralized moss samples before and after the sorption process. A 0.4 g sample of moss was taken and dried at 323 K were used for mineralization.

Mosses were collected at various locations in Bory Stobrawskie. The main condition that had to be met by a location was the occurrence of at least three species of tested mosses per 1 m^2^. After purification from any mechanical impurities mosses were washed with demineralised water. After drying at 323 K, mosses were mineralized. The concentrations of Zn and Cd were determined in the mineralized samples.

In determination of heavy metals concentrations, atomic absorption spectrometer SOLAAR 969 (UNICAM) was used, manufactured by Thermo Electron Corporation, USA. ANALYTIKA Ltd. (the Czech Republic) standards were used to calibrate the equipment. In spectrometer calibration, standards produced by ANALYTIKA Ltd. (the Czech Republic) were used. The largest concentrations of standard solutions used for calibration: 5 mg dm^−3^ for Zn and 2 mg dm^−3^ for Cd were used as the limit values of a linear relationship between the signal and concentration. Where appropriate, samples used for metal determination were diluted below these values. Mosses were mineralized using the CEM’s MARS-X microwave system. Solutions were prepared using MERCK’s reagents.

Figures 2 and 3 were taken by means of the Nikon’s SMZ 1500 DIA/EPI stereoscopic microscope with the digital image recording feature, using the NIS-Elements Basic Research software. Images were recorded using a video camera.

The limits of detection and determination of the analyzed metals were, respectively, as follows: Zn (0.0033 and 0.013 mg dm^−3^) and Cd (0.0028 and 0.032 mg dm^−3^). The quality control of measurements was assured by test analyses of the BCR 414 plankton and BCR-482 lichen reference materials from the Institute for Reference Materials and Measurements in Belgium. The obtained results are summarized in Table 1.

**Table 1:** Measured and certified values of Zn and Cd concentration in the BCR 414 plankton and BCR 482 lichen reference material

Metal	BCR 414 plankton					BCR 482 lichen				
	*Cv*	±*U*	AAS		*D *(%)	*Cv*	±*U*	AAS		*D *(%)
			Mean	±*SD*				Mean	±*SD*	
	(μg g^−1^)					(μg g^−1^)				
Zn	103	4	112	3	−8.0	93.9	2.5	100.6	2.2	−6.7
Cd	n.d.	n.d.	0.383	0.014	n.d.	0.52	0.04	0.56	0.02	−7.1

*Cv* certified value, *U* uncertainty, *SD* standard deviation, *D* deviation: the relative difference between measured by AAS and certified concentrations in %

To describe the equilibrium, the Langmuir isotherm model was used:

1 $$ \frac{1}{{c_{{M\left( {m,1} \right)}} }} = \frac{1}{{c_{{M\left( {m,max} \right)}} }} + \frac{1}{{c_{{M\left( {m,max} \right)}} \cdot K}} \cdot \frac{1}{{c_{{M\left( {s,1} \right)}} }} $$

where: *c*_*M*_ is the metal concentration in mosses (*m*) or in the solution (*s*) in the equilibrium state (1), the index (*max*) denotes the sorption capacity of mosses, *K* – the Langmuir partition coefficient.

Conversion of the determination results of metals concentrations in mineralized moss samples require some additional explanation. The authors point out that both, the ion-exchange process kinetics (Gupta et al. 2006) and the equilibrium parameters in a static system (Gupta et al. 2006; Rajfur et al. 2012) depend on the mass of the biosorbent used in a given solution volume.

On base of Eq. (1) the isotherms were determined separately for each moss species. Concentrations of metals were determined in starting solution and in moss after mineralization. The isotherm common for all moss species investigated (7 g of total mass) was also determined using the data from Table 2.

**Table 2:** Measured metal cation concentrations (mg dm^−3^) in solutions before (*c*_*0*_) and after (*c*_*1*_) the sorption process

Zn				Cd			
*c*_0_	±*SE*_*C*0_	*c*_1_	±*SE*_*C*1_	*c*_0_	±*SE*_*C*0_	*c*_1_	±*SE*_*C*1_
–	–	–	–	221	11	166.3	8.1
62.3	2.8	36.5	1.7	109.2	4.5	69.3	3.3
33.5	1.3	11.85	0.46	57.3	2.5	21.9	1.0
18.21	0.62	2.38	0.10	30.8	1.1	5.44	0.22
9.05	0.35	0.537	0.022	15.60	0.58	2.79	0.11
4.01	0.14	0.1731	0.0075	6.19	0.21	0.800	0.031

*SE* standard error

It occurs particularly in the case of low initial concentrations in solution, and is related to the quantity and type of cations desorbed from the biomass. Therefore, the following formula was adopted to assess the sorption properties of the analyzed moss species:

2 $$ c{\prime }_{{M\left( {m,1} \right)}} = 7 \cdot c_{{M\left( {m,1} \right)}} $$

where: *c’*_*M*(*m*,*1*)_ – metal concentration in 7 g of mosses in the equilibrium state.

This interpretation makes it possible to keep the proportion of the volume of solutions and the mass of mosses used for experiments. Hypothetically, the sorption process in seven species of the analyzed mosses, 1 g each, was replaced by a single 7 g species. The validity of these premises was confirmed by the results shown below.

## Results and Discussion

Table 2 shows the determined concentrations of metal cations in solutions before (*c*_0_) and after (*c*_1_) the sorption process and, their standard error values ±*SE* (mg dm^−3^). The pH indicator of the solution during the experiment varied within the limits of 4.7–4.1, which protects against precipitation of the hydroxides of the metals studied.

As shown in the Table 2, the uncertainty of measurements expressed by the standard error does not exceed ±5 % of the mean value. For comparison, standard errors of the results of Zn and Cd determination in the mineralized moss samples did not exceed ±8.7 % and ±11.4 %.

**Fig. 1:**
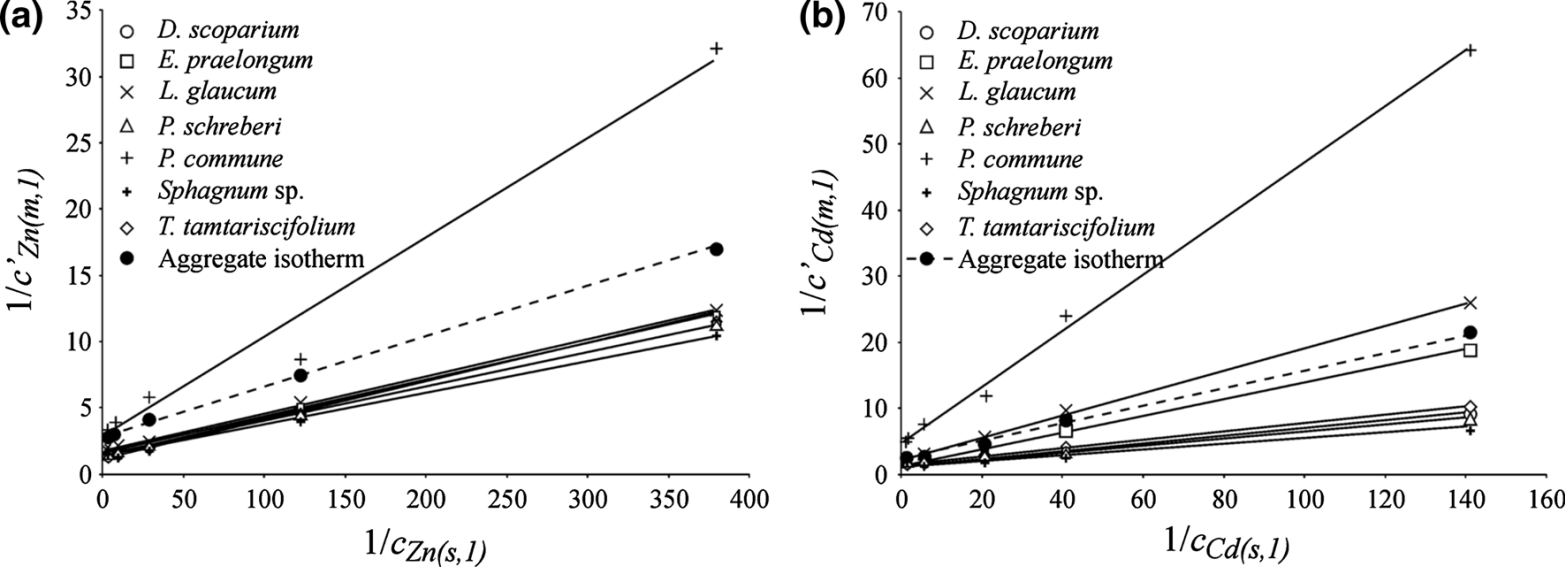
Sorption isotherms; **a** Zn, **b** Cd

In order to further interpret the results, concentrations were expressed in mmol dm^−3^ or in mmol g^−1^ d.m. The diagrams in Fig. 1a, b show Langmuir isotherms (relationship 1) for Zn and Cd, taking into account relationship 2.

Table 3 includes parameters of straight line fits shown in Fig. 1a, b. The Table also shows moss sorption capacities calculated per 1 g of dry matter (*c*_*M(m,max*_ *)*). The mean of parameter *b* and the mean of parameter *a* were calculated using values of these parameters determined separately for seven analyzed moss species. It should be reminded that the aggregate isotherm parameters were determined on the basis of initial and final concentrations of the analyzed metals in solutions (Table 2).

**Table 3:** Parameters of the Langmuir isotherm *y* = *b·x* + *a*, where: *b* = 1/(*c’*_*M*(*m*,*max*)_·*K*), *a* = 1/*c’*_*M*(*m*,*max*)_, *SE* standard error, *R* ^2^ coefficient of determination, *c*_*M*(*m*,*max*)_ sorption capacity of 1 g of moss dry matter [mmol (g d.m.)^−1^]

Moss species	*b*	±*SE*_*b*_	*a*	±*SE*_*a*_	*R* ^2^	*c*_*M*(*m,max*)_	*K*
Zn							
*D. scoparium*	0.0277	0.0023	1.33	0.42	0.981	0.1070	48.0
*E*. *praelongum*	0.0293	0.0011	1.11	0.19	0.996	0.1287	37.9
*L*. *glaucum*	0.0349	0.0026	1.523	0.082	0.997	0.0821	43.6
*P*. *schreberi*	0.02587	0.00063	1.47	0.11	0.998	0.0972	56.8
*P*. *commune*	0.0750	0.0077	2.9	1.4	0.969	0.0491	38.7
*Sphagnum* sp.	0.02379	0.00065	1.40	0.12	0.998	0.1020	58.8
*T*. *tamtariscifolium*	0.02786	0.00068	1.59	0.12	0.998	0.0898	57.1
Mean (*me*)	0.0349	0.0016	1.62	0.28	–	0.0938	46.4
Aggregate isotherm (*ai*)	0.0379	0.00093	2.83	0.17	0.998	0.0505	74.7
Cd							
*D*. *scoparium*	0.0590	0.0026	1.07	0.16	0.992	0.1335	18.1
*E*. *praelongum*	0.1276	0.0028	1.19	0.17	0.998	0.1200	9.3
*L*. *glaucum*	0.1696	0.0044	2.15	0.27	0.997	0.0664	12.7
*P*. *schreberi*	0.0534	0.0013	1.126	0.080	0.998	0.1264	21.1
*P*. *commune*	0.425	0.020	4.5	1.2	0.996	0.0319	10.6
*Sphagnum* sp.	0.04393	0.00072	1.192	0.043	0.999	0.1200	27.1
*T*. *tamtariscifolium*	0.0635	0.0015	1.44	0.089	0.999	0.0992	22.7
Mean (*me*)	0.1345	0.0040	1.81	0.24	–	0.0997	13.5
Aggregate isotherm (*ai*)	0.1329	0.0055	2.39	0.33	0.993	0.0597	18.0

Comparison of the mean value of the slope *b*_*me*_ and the absolute term *a*_*me*_ to the values of these parameters determined in relation to the aggregate isotherm (*b*_*ai*_ and *a*_*ai*_) is an important element of the research. The difference between two independent values *b*_*me*_ and *b*_*ai*_ (the common element is the concentration of the solution in equilibrium: *c*_*M(s,1)*_) compared to the mean value of *b* does not exceed 8 % for Zn and 2 % for Cd. For comparison, parameter *a*, which denotes the sorption capacity of mosses, differs by approximately 42 % and 24 %, respectively. Furthermore, the standard errors of parameters *b* determined separately for seven moss species (max 10 %) are several times smaller than the standard errors of parameter *a* (max 48 %). These data show that the assessment of the sorption capacity based on the Langmuir isotherm (*a* = 1/*c*_*M*(*m*,*max*)_) can be subject to a significant error.

In the case of Zn, the determined sorption capacity changes depending on the moss species from 0.0491 to 0.1287 mmol (g d.m.)^−1^ and in the case of Cd from 0.0319 to 0.1335 mmol (g d.m.)^−1^. As mentioned above, the uncertainty reaches the value of 48 %. For comparison, the determined sorption capacity of *H. splendens* in the case of Cd is 0.289 mmol g^−1^ d.m. (Sari et al. 2008) and the sorption capacity of *F. antipyretica* aquatic mosses is 0.229 mmol (g d.m.)^−1^ in the case of Zn and 0.249 mmol (g d.m.)^−1^ the case of Cd (Martins et al. 2004).

As shown in Table 3, much less uncertainty of measurements is observed in the case of the slopes of the lines: *b* = 1/(*c*_*M*(*m*,*max*)_·*K*). Assuming that *c*_*M*(*m*,*max*)_ is constant and that *K* ~ *c*_*M*(*m*,*1*)_/*c*_*M*(*s*,*1*)_, as the sorption preferences of mosses increase, when *c*_*M*(*m*,*1*)_ increases, the value of *b* decreases. Therefore, taking into account the standard error indicating statistically significant differences in the slopes, it can be concluded that the sorption of Zn and Cd depends on moss species and increases in the series: *P. commune* < *L. glaucum* < *Eurhynchium praelongum*  < *T. tamtariscifolium* ≤ *D. scoparium* ≤ *P. schreberi* < *Sphagnum* sp.

**Fig. 2:**
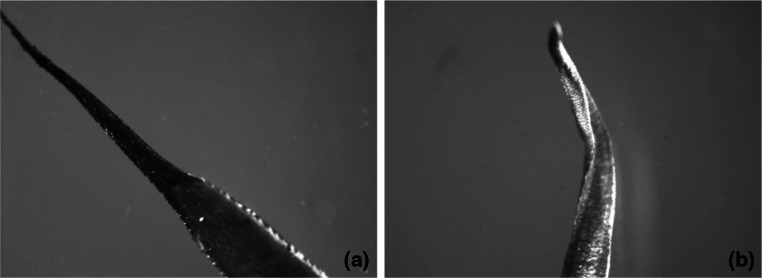
Moss leaves: **a** *P. commune*, **b** *L. glaucum*

**Fig. 3:**
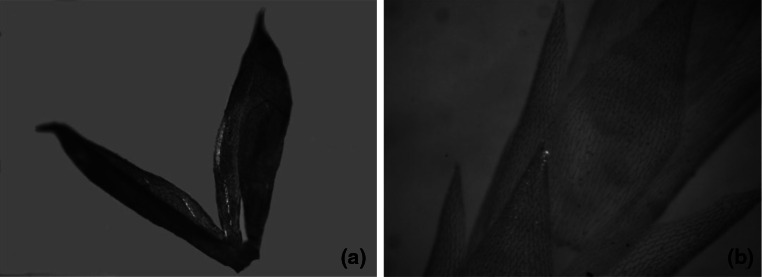
Moss leaves: **a** *P. schreberi*, **b** *Sphagnum* sp

Figures 2 and 3 show the surface of leaves of two moss species demonstrating the lowest heavy metal sorption properties and of two moss species demonstrating the highest heavy metal sorption properties.

Images in Figs. 2 and 3 show clear differences in the development of the moss surface. The leaves of *D. scoparium* and *P. schreberi* (Fig. 3), because of their trough-like shape, have a larger surface area in proportion to their mass as compared to thick and poorly developed leaves of *P. commune* and *L. glaucum* shown in Fig. 2. Taking into account the results of research presented earlier, it can be assumed that the sorption capacity of mosses increase with the development of their surface area, which in turn points to the surface location of active centers involved in the ion-exchange process.

The next stage of the experiment involved checking whether in the natural conditions the moss species absorb the tested metals in proportions determined experimentally in the laboratory. The measured concentrations of Zn and Cd in different moss species collected from the area of soil not exceeding 1 m^2^ were compared to the concentrations of tested metals in *P. schreberi* commonly found in the area covered by the research. To this end, the slopes of lines listed in Table 3 were compared:

3 $$ c^{ * }_{M} = \frac{{b_{\text{Moss}} }}{{b_{{{\text{P}} . {\text{schreberi}}}} }} \cdot c_{M} $$

where: c*_M_ – reduced value, *b*_Moss_ – the slope of the line specific to a given moss species, *b*_P.schreberi_ – the slope determined for *P. schreberi*, *c*_*M*_ – concentration of metal in mosses.

Table 4 shows sample mean values of concentrations *c*_*M*(*me*)_ and mean values of reduced concentrations c*_M(me)_ determined for three moss species and the appropriate *SE* values.

**Table 4:** *c*_*M*(*me*)_ and c*_M(*me*)_ [mmol (kg d.m.)^−1^] as well as *SE* and SE* parameters determined for various moss species collected from one location

Moss species	Metal	*c*_*M*(*me*)_	±*SE*	c*_M(*me*)_	±SE*
*P. schreberi*, *P. commune*, *Sphagnum* sp.	Zn	0.50	0.37	0.52	0.28
	Cd	0.027	0.018	0.040	0.011
*D. scoparium*, *P. schreberi*, *T. tamtariscifolium*	Zn	1.06	0.18	1.10	0.14
	Cd	0.033	0.010	0.035	0.007
*P. schreberi*, *P. commune*, *T. tamtariscifolium*	Zn	0.91	0.55	1.44	0.76
	Cd	0.044	0.025	0.089	0.048

The results presented in the Table show that the reduced concentrations of tested metals accumulated in various moss species collected from one location are characterized by a smaller dispersion of results: SE* < *SE* but still high values of SE* indicate the influence of other, unrecognized biotic and abiotic factors affecting the level of heavy metal accumulation in mosses. The ability to accumulate dry deposition containing heavy metals in troughs (Fig. 3) or in the porous surface of mosses can be one of these factors. The diffusion of pollutants from soil in the layer of water that wets the mosses is also important (Kłos et al. 2012).

## Conclusions

The results of this research indicate that one of the factors affecting the sorption properties of mosses is the proportion of the surface area to their mass, which in turn points to the surface location of active centers involved in the ion-exchange process. The field studies showed that this is not the only factor affecting the accumulation of heavy metals in mosses in natural conditions. The presented results can play a significant role in the process of planning and interpreting the results of biomonitoring research.
